# Aerobic Exercise Improves Cognitive Recovery in Mice with Chronic Cerebral Hypoperfusion by Modulating the Annexin-A1-MAPK Axis and Astrocyte Polarization

**DOI:** 10.14336/AD.2024.01213

**Published:** 2025-12-14

**Authors:** Wei Zhang, Jing He, Yuxin Wang, Xiaozhen Wang, He Jin, Xu Zhang, Ling Kong, Yanchuan Wu, Yong Yang, Rong Wang

**Affiliations:** ^1^Central Laboratory, Xuanwu Hospital, Capital Medical University, Beijing, China.; ^2^Beijing Geriatric Medical Research Center, Beijing, China.; ^3^Beijing Institute of Major Brain Diseases, Beijing, China.; ^4^School of Chinese Medicine, Beijing University of Chinese Medicine, Beijing, China.

**Keywords:** Vascular cognitive impairment, aerobic exercise, Annexin-A1, MAPK, astrocytes polarization, synapse plasticity

## Abstract

Vascular cognitive impairment and dementia (VCID), resulting from chronic cerebral hypoperfusion, represent the second most prevalent form of dementia globally. Aerobic exercise is widely acknowledged as an effective intervention for various cognitive disorders. This study utilized a bilateral common carotid artery stenosis (BCAS) model to investigate whether aerobic exercise promotes cognitive recovery through the Annexin-A1 (ANXA1)/mitogen-activated protein kinase (MAPK) axis in BCAS mice. Our findings demonstrate that aerobic exercise improved spatial memory in BCAS mice by enhancing white matter (WM) integrity and hippocampal function. WM integrity was confirmed through Luxol Fast Blue (LFB) staining and protein assays. Additionally, aerobic exercise mitigated BCAS-induced long-term potentiation (LTP) decay and upregulated hippocampal expression of key synaptic proteins, including N-methyl-D-aspartate receptor subunits NR2B and NR1, vesicular glutamate transporter 1 (vGluT1), and the synaptic scaffolding protein postsynaptic density protein 95 (PSD95). Furthermore, aerobic exercise enhanced the expression of the anti-inflammatory mediator ANXA1 through exosome secretion while simultaneously suppressing the MAPK signaling pathway. These molecular changes were associated with increased astrocyte proliferation and the polarization of astrocytes toward the A2 phenotype. These findings were further validated using an in vitro co-culture model of astrocytes (U251) and neurons (HT22). In summary, our study demonstrates that aerobic exercise improves WM integrity and hippocampal function by modulating the ANXA1/MAPK axis following astrocyte polarization. Thus, aerobic exercise emerges as a promising intervention for promoting functional recovery in VCID.

## INTRODUCTION

Vascular cognitive impairment and dementia (VCID) is the second most prevalent form of dementia globally [1]. It is estimated that up to 60% of stroke survivors experience varying degrees of cognitive impairment, ranging from mild to severe, within the first year following a stroke [2]. The risk of VCID increases with the progression of stroke severity [3, 4]. Chronic cerebral hypoperfusion is a key pathological feature of VCID, leading to significant neuronal challenges [5, 6]. Given the rising prevalence of VCID, there is an increasing focus on developing effective treatment and preventive strategies.

Chronic cerebral hypoperfusion results in astrocyte proliferation, white matter (WM) damage, and impaired hippocampal plasticity, all of which contribute to cognitive dysfunction [7, 8]. These changes disrupt neural connectivity and function. WM lesions and hippocampal dysfunction are key pathological features of chronic cerebral ischemia [9, 10]. Consequently, improving WM integrity and enhancing hippocampal synaptic plasticity are considered effective therapeutic strategies. However, the exact mechanisms underlying these therapeutic strategies are still unclear, highlighting the need for further research.

Several clinical studies have demonstrated that regular aerobic exercise reduces the risk of developing VCID [11, 12]. Exercise enhances cognitive function by increasing neurotrophin uptake, promoting endothelial function, and modulating vascular risk factors [13, 14]. Additionally, exercise plays a crucial role in regulating neuroinflammation by modulating the secretion of cytokines [15], neuropeptides, and other factors [16], suggesting that aerobic exercise may influence VCID by altering neuroinflammatory responses, however, the precise mechanisms require further investigation.

Astrocytes, central to the neuroinflammatory response, are the most abundant type of neuroglial cells [17]. Normally, astrocytes remain in a resting state, but following ischemic and hypoxic stimuli, they undergo morphological and functional changes, transforming into reactive astrocytes [18]. These reactive astrocytes are classified into two phenotypes: A1 and A2. A1 astrocytes impair neuronal function, while A2 astrocytes release neurotrophic factors that support neuronal repair [19]. Beyond maintaining homeostasis, astrocytes also play critical roles in synapse formation, synaptic plasticity, and regulating cognitive functions related to synapses [18, 20]. In VCID, a neuroinflammation-induced reduction in hippocampal synaptic plasticity is a key pathological process in the disease’s onset and progression [21], underscoring the importance of understanding how astrocytes respond to chronic cerebral blood flow reduction and influence cognitive functions.

Annexin-A1 (ANXA1) is a member of the membrane-bound protein superfamily that binds phospholipids in a calcium-dependent manner. ANXA1 plays a variety of physiological roles, including contributing to the structural composition of cells, regulating growth, and facilitating vesicular transport [22]. Recent studies have shown that ANXA1 effectively mitigates central inflammatory damage caused by cerebral ischemia-reperfusion by promoting the polarization of microglia toward the M2 phenotype, which is beneficial for healing and repair [23]. The hyperactivation of the mitogen-activated protein kinase (MAPK) signaling pathway promotes immune response activation following ischemic insults, while ANXA1 plays a regulatory role in immune injury [24]. Importantly, ANXA1 has been shown to induce the aggregation of exosomes, and exosomes, in turn, influence ANXA1 expression [25].

Exosomes are extracellular vesicles with diameters ranging from 30 to 200 nm that carry critical molecular information, including mRNA, microRNA (miRNA), and proteins [26]. They play a key role in cell-to-cell communication and messaging [27]. Due to their ability to target specific receptors and be efficiently internalized by recipient cells, exosomes have emerged as potential tools for delivering treatments for brain diseases [28]. Studies have shown that exosomes are involved not only in the transfer of toxic proteins during disease processes but also in delivering beneficial proteins for therapeutic purposes [29].

In summary, this study aimed to investigate whether aerobic exercise could improve WM and hippocampal function by modulating astrocyte-mediated central immune responses in mice with chronic cerebral hypoperfusion. Additionally, it sought to elucidate the role of exosome-mediated ANXA1/MAPK axis immune mechanisms in this process. These studies aim to offer novel insights and strategies for the treatment of VCID, potentially impacting clinical practices and paving the way for future research.

## MATERIALS AND METHODS

### Animals

Male C57BL/6 mice, aged 6-8 weeks and weighing 22-25 g, were obtained from Beijing Vital River Laboratory Animal Technology Co. Ltd. The experimental design and protocols for the animal experiments received approval from the Ethical Review Committee of Xuanwu Hospital, Capital Medical University. Mice were housed in temperature- and humidity-controlled rooms maintained at 24-26°C and 50%-55% humidity, with unrestricted access to food and water. All surgeries were performed under anesthesia to minimize pain and discomfort to the animals.

### Bilateral common carotid artery stenosis (BCAS) model

BCAS model was performed, as previously described [30]. Mice were anesthetized and positioned supine, with body temperature maintained at 36.5 ± 0.5°C using a heating pad. A midline incision was made in the neck, exposing the common carotid artery. A 0.18-mm-diameter spring microcoil (Sawane Spring Co., Japan) was wound around one common carotid artery, followed by the same procedure on the other artery 30 min later. In the Sham group, an identical procedure was performed, but no coils were placed.

### BrdU injection

Each group of mice received daily intraperitoneal injections of BrdU at a dose of 50 mg/kg for 7 consecutive days following BCAS surgery. BrdU expression was subsequently assessed by immunofluorescence.

### Experimental design and treadmill running protocol

Male C57/BL6 mice were randomly assigned to three groups: Sham, BCAS, and EX (BCAS + Exercise). Both the BCAS and EX groups underwent BCAS surgical modeling. On the fourth day following BCAS modeling, the EX group began treadmill running training. The first 2 days served as an adaptation phase, with a treadmill speed of 10 m/min for 30 min per day, including a 3 min rest every 10 min. Starting on the third day, the treadmill speed was increased to 15 m/min, and the mice ran for 30 min daily, 5 days per week, for 2 months. The Sham and BCAS groups were placed on the treadmill at the same time each day, but the treadmill was not activated.

### Tissue sample collection

The cerebral hippocampus and WM were promptly dissected on ice and rapidly frozen in liquid nitrogen (*n* = 7). For histological analysis, mice were deeply anesthetized and perfused with 30 mL of cold 1 × PBS, followed by 30 mL of cold 4% paraformaldehyde. After perfusion, the brains were extracted, placed in vials containing 4% paraformaldehyde, and subjected to sucrose gradient dehydration and embedding (*n* = 5).

### Extraction of synaptosomes

Extraction of synaptic vesicles as described [31]. Hippocampal tissues were homogenized in HEPES buffer containing 0.32 M sucrose and centrifuged at 600 × g for 10 min. The supernatant was diluted with HEPES buffer to a final sucrose buffer concentration of 800 mM, and centrifuged at 1200 × g for 15 min. The supernatant was discarded, and the impurities were washed away by centrifugation with HEPES buffer at 12000 × g for 15 min. Synaptic vesicles were used for subsequent synaptic protein detection experiments.

### Y-maze test

The Y-maze experiment was conducted to assess the spatial exploration abilities of mice, as previously described [32]. The apparatus consists of three arms (34 × 8 × 14 cm) arranged at 120° angles. The test included two trials, spaced 1 h apart. In the first trial, one arm (the novel arm) was closed off, and the mice were allowed to explore the other two arms freely for 5 min. In the second trial, the mice were placed back in the starting arm with free access to all three arms for 5 min. Arm entry was defined as all four paws entering the arm. The total time spent by the mice in the novel arm was recorded and analyzed (*n* = 15).

### Novel object recognition (NOR) test

The NOR test assesses non-spatial recognition memory in mice [33] and is conducted over three days. On the first day, mice undergo a habituation phase where they freely explore an open field (50 cm × 50 cm × 25 cm) for 10 min. On the second day, during the training phase, two identical objects are placed in opposite corners of the open field, and the mice are allowed to explore them freely for 10 min. The open field is cleaned with 70% alcohol afterward to eliminate olfactory cues. On the third day, during the test phase, one object is replaced with a novel object, and the mice are reintroduced to the field to explore for 5 min. The time spent and the number of interactions with each object are recorded (*n* = 15). Results are expressed as a discrimination index calculated as: (time novel - time familiar)/(time novel + time familiar).

### Morris water maze (MWM) test

The MWM test was used to evaluate spatial learning and memory abilities in mice [34]. The apparatus consisted of a 120 cm diameter pool, evenly divided into four quadrants, with a platform located in the center of the fourth quadrant. For 5 days, the platform was submerged 1-2 cm below the water surface, and mice were introduced to the maze from different quadrants, given 2 min to find the platform. The time taken to locate the platform was recorded as the latency period. Mice that failed to find the platform within 2 min were guided to it by the experimenter and remained on the platform for 30 seconds. On the sixth day, following 5 days of training, the platform was removed for a 2 min exploration test. Data such as the latency period and the number of times the platform location was crossed were automatically recorded (*n* = 15).

### Open field test

Open field test as previously described [35]. The Open Field Test was conducted in an open field apparatus measuring 50 cm × 50 cm × 25 cm. The floor of the field was divided into 16 equal squares, with the central 4 squares designated as the central area. Mice were acclimated to the experimental environment for 30 min and then allowed to familiarize themselves with the apparatus for 5 min. During the test phase, mice were placed in the center, and a video analysis system recorded their trajectory for 10 min. Data collected included total distance traveled, distance traveled in the central area, and speed (*n* = 15).

### Balance beam test

The Balance Beam Test is utilized to evaluate locomotion and balance in mice [36]. Mice were placed at one end of a balance beam (100 cm in length, 2 cm in width, and 40 cm in height), with a dark-colored door frame (10.5 × 10.5 × 10.5 cm) positioned at the opposite end. During the first three days, mice were trained to traverse the beam without turning or pausing. On the fourth day, the balance beam test was conducted, and the time taken to cross the beam, as well as the number of slips, were recorded (*n* = 15).

### Two-dimensional laser speckle imaging

Cerebral blood flow (CBF) was measured in mice using a laser scatter contrast analysis system, as per protocols previously described [37]. Mice were anesthetized and their body temperature maintained at 36.5 ± 0.5°C. A midline incision was made in the scalp to expose the skull, from which CBF was recorded at a distance of 10 cm above the skull (*n* = 6).

### Electrophysiological recording

As previously described [31], field excitatory postsynaptic potentials (fEPSPs) were recorded using MED64 planar microelectrodes (Panasonic, Osaka, Japan). Briefly, mice were decapitated, and their brains were quickly removed and immersed in chilled artificial cerebrospinal fluid (ACSF). Coronal sections, 400 μm thick, were prepared using a vibrating slicer and then incubated at room temperature for 30 minutes. Under a microscope, the stimulating electrode was positioned on the schaffer collateral pathway in the hippocampal CA3 region, while the recording electrode was placed in the CA1 region's pyramidal cell layer. The stimulus intensity was adjusted to achieve fEPSP peak amplitudes at half of their maximum value. Recordings were taken for 30 minutes to establish a baseline. Long-term potentiation (LTP) was induced using theta-burst stimulation (TBS), consisting of bursts at 10-second intervals. Each burst included four pulses at 100 Hz, with a 200 ms inter-pulse interval. fEPSPs were monitored for at least 60 minutes post-LTP induction (n = 10 slices from 5 mice).

### Golgi stain

The experiments were conducted following the instructions provided with the FD Rapid GolgiStain™ Kit (FD NeuroTechnologies, USA). In brief, after anesthesia, approximately 8 mm of mouse coronal sections, including the hippocampus, were immersed in a dip solution consisting of equal parts of Solution A and Solution B. The tissues were kept at room temperature in the dark for two weeks. Subsequently, the tissue was transferred to Solution C and stored at room temperature in the dark for one week. The tissue was then sliced into 100 μm sections using a freezer sectioning machine and air-dried at room temperature. Finally, the sections were stained, dehydrated, and sealed with neutral resin. Synaptic images of hippocampal neurons were captured under a light microscope, and the number of dendritic spines was counted (*n* = 5).

### Luxol fast blue (LFB) and cresyl violet staining

Coronal sections (4 μm) were prepared and stained with LFB to assess the severity of WM damage. Briefly, sections were immersed in LFB solution (Abcam, UK) for 18-24 hours. Excess stain was removed using 95% ethanol, followed by a 20-second treatment with 0.05% lithium carbonate aqueous solution (Abcam, UK) and 70% ethanol until the sections were decolorized. Subsequently, sections were immersed in polyethylene violet solution (Abcam, UK) for 5 minutes and washed with deionized water. After dehydration through an ethanol gradient, sections were cleared in xylene and mounted with neutral resin.

Five brain regions were evaluated for WM damage: medial corpus callosum, paramedial corpus callosum, caudoputamen, optic tract, and internal capsule. WM injury severity was classified into four grades: normal (grade 0), nerve fiber disorganization (grade 1), formation of noticeable vacuoles (grade 2), and loss of myelinated fibers (grade 3). Additionally, the number of neurons in the hippocampal CA1, CA2, and CA3 regions was counted to assess neuronal integrity (*n* = 5).

### Exosome extraction and characterization

The exosome extraction procedure followed established protocols [38]. Fresh brain tissue was washed with 1×PBS. Two drops of RPMI-1640 were added to a 1.5 ml EP tube, and the tissue was minced with micro-scissors. Between 1.5 ml and 2 ml of digestive solution was added, and the mixture was incubated for 1 h at 37°C in a shaking incubator. The digestive solution was then filtered through a 70 μm filter. The filtrate underwent differential centrifugation at 300 × g, 2000 × g, 10,000 × g, and 120,000 × g. After centrifugation, 100-200 μl of the final suspension was collected, and the pellet was resuspended in 1×PBS. Exosome samples were identified by nanoparticle tracking analysis (NTA), transmission electron microscopy (TEM), and detection of the protein marker tumor susceptibility gene 101 (TSG101).

### Co-culture and treatment of HT22 and U251 cells

The HT22/U251 co-culture was conducted as previously described [39]. U251 cells (2 × 10^5^) were seeded on a permeable insert with a pore size of 0.4 μm (Corning, NY, USA), positioned above HT22 cells (1 × 10^5^) in a 24-well plate. The experiment was divided into four groups: Mock (PBS), GW4869 (GW4869), Exos (NC-Exos) and EX-Exos (Exercise-Exos). Following 12 hours of co-culture, LPS (1 μM) was added to the GW4869, Exos and EX-Exos groups for a 20-hour intervention. After the 20-hour incubation, LPS was removed. The GW4869 group was treated with GW4869 (10 μM), the Exos group with NC-Exos (10 μg/μl), and the EX-Exos group with Exercise-Exos (10 μg/μl) for 24 hours. The Mock group received equal volumes of PBS. The culture supernatant was collected for ELISA analysis, while U251 cells underwent protein quantification.

### Western blotting

The experimental methods, as previously described [40], involved protein extraction from isolated tissue samples of the hippocampus, cortex, and WM. The extracted proteins were separated using sodium dodecyl sulfate-polyacrylamide gel electrophoresis (SDS-PAGE) and subsequently transferred to membranes through electroporation. Immunoblotting was conducted using a variety of primary antibodies, including ANXA1 (Abcam, ab137745), BrdU (Abcam, ab8152), C3 (Abcam, ab200999), MAPK (Cell Signaling, 9102s), glial fibrillary acidic protein (GFAP) (Cell Signaling, 3670), HRAS (Proteintech, 18295-1-ap), Iba1 (Abcam, ab178847), myelin basic protein (MBP) (Abcam, ab40390), neurofilament 200 (NF200) (Sigma, N5389), NR1 (Abcam, ab134308), NR2A (Abcam, ab65783), NR2B (Sigma, SAB4501304), p53 (Cell Signaling, 2524s), phosphorylated MAPK (p-MAPK) (Cell Signaling, 4370s), and S100A10 (Invitrogen, PA5-95505). The protein bands were ultimately detected using chemiluminescence, with each experiment conducted in replicates of seven (*n* = 7).

### Immunofluorescence staining

The method followed previously established protocols. Coronal sections with a thickness of 10 μm were antigenically repaired and then membrane-broken with 0.25% Triton-X100. 1% BAS was used to seal the sections after overnight incubation at 4°C with primary antibody as shown above. Next day fluorescent secondary antibody was incubated at room temperature for 1 h. Sections were blocked with DAPI-containing blocking agent. Positive cells were obtained from different regions of the microscope according to the experimental protocol, ensuring consistent region selection between and within groups (*n* = 5).

### Enzyme-linked immunosorbent assay (ELISA)

The levels of inflammatory cytokines (IL-1β, IL-4, IL-10, TNF-α) in mouse serum were measured using a double-antibody one-step sandwich ELISA method, as described previously [41]. The assay was performed strictly in accordance with the instructions provided with the ELISA detection kit from NeoBioscience Technology, China.

### Statistical analysis

Data were expressed as means ± standard deviations. Statistical comparisons between two groups were performed using Student's t-test, while differences among multiple groups were assessed using one-way ANOVA followed by Bonferroni/Dunn post hoc correction. Behavioral data were analyzed using the nonparametric Kruskal-Wallis test to determine differences between groups. Pearson correlation analysis was employed to evaluate the association between ANXA1 expression and cognitive function. All statistical analyses were conducted using SPSS version 22.0, and a p-value of less than 0.05 was considered statistically significant. The experiments were carried out under a double-blind design to ensure objectivity and reduce bias. Data analysts were kept unaware of the treatment conditions assigned to different groups during both the execution of the experiments and the analysis of results. Each mouse was assigned a unique identifier consisting of a single letter, and handling was restricted to designated experimental personnel. The specific group assignments were disclosed to the data analysts only after the completion of the data analysis.

## RESULTS

### Aerobic Exercise Enhances Spatial Memory in BCAS Mice

**Figure 1.**
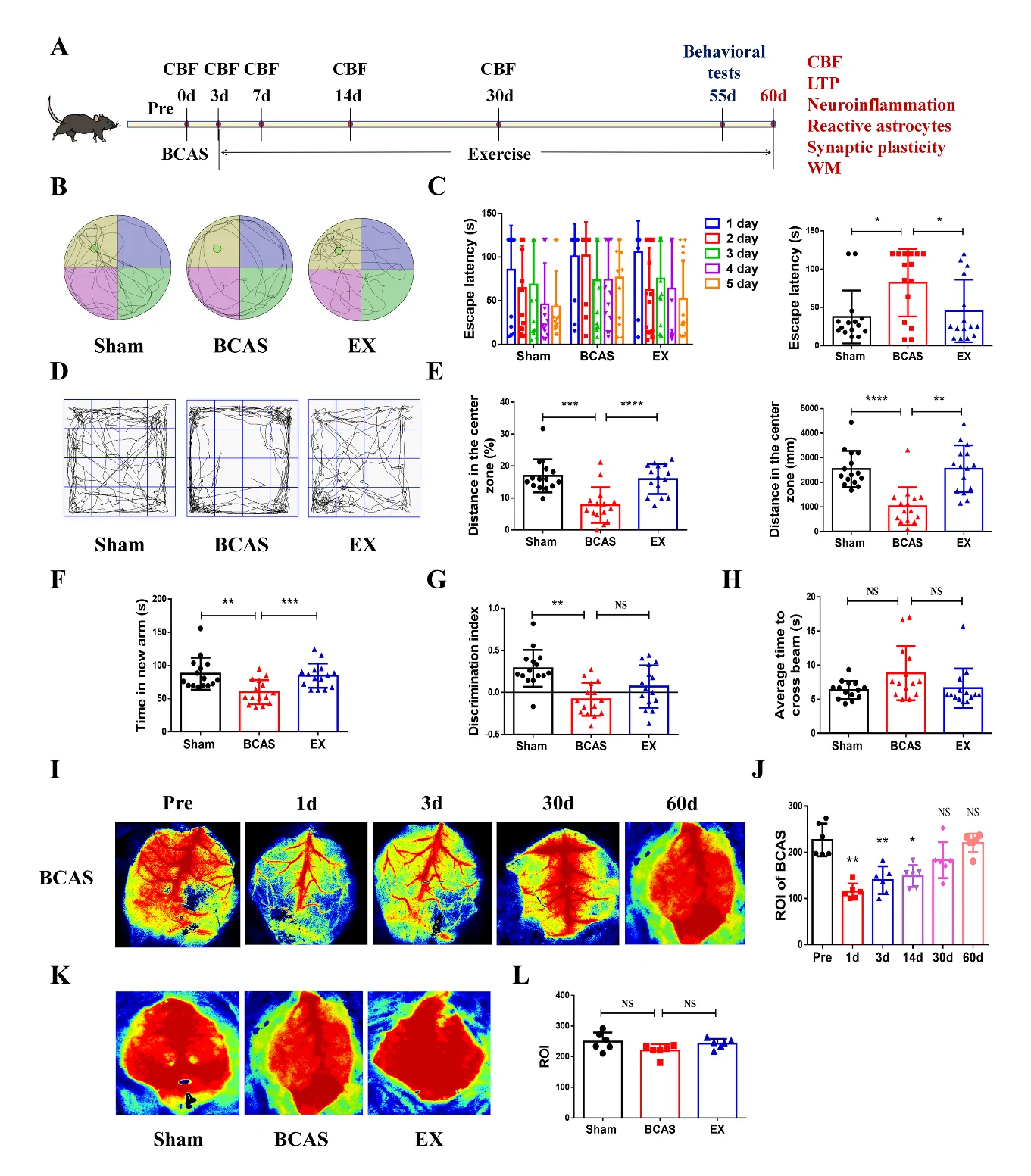
**Aerobic exercise enhances spatial memory capacity in BCAS mice**. (**A**) Overview of the study design. (**B**) Representative images of Morris Water Maze (MWM) motor trajectories. (**C**) Escape latency measured during 5 consecutive days of MWM training and escape latency after platform removal on the test day. (**D**) Representative trajectory images from the open-field experiment. (**E**) Distance traveled and percentage of time spent in the center region during the open-field experiment. (**F**) Time spent exploring new arms in the Y-maze. (**G**) Discriminant index from the novel object recognition test. (**H**) Traversal time on the balance beam. n = 15 mice per group. (**I**) Schematic representation of cerebral blood flow (CBF) at various time points following BCAS modeling. (**J**) Quantitative analysis of CBF at different time points post-modeling. (**K**) Schematic diagram of CBF at 2 months after modeling in the three experimental groups. (**L**) Quantitative analysis of CBF at 2 months post-modeling across the three groups. Bar graphs are presented as mean ± SD. n = 6 mice per group. NS: no significance. *p < 0.05, **p < 0.01, ***p < 0.001.

The classical BCAS model simulated VCI due to chronic cerebral hypoperfusion. Cognitive function was assessed using the MWM, Y-maze, open field, and NOR tests. The BCAS group exhibited increased escape latency in the MWM (p < 0.05, Fig. 1B, C), suggesting hippocampal dysfunction, and reduced exploration in the Y-maze and open field tests (p < 0.01, Fig. 1D, E, F), indicating impaired spatial memory and exploratory behavior.

**Figure 2.**
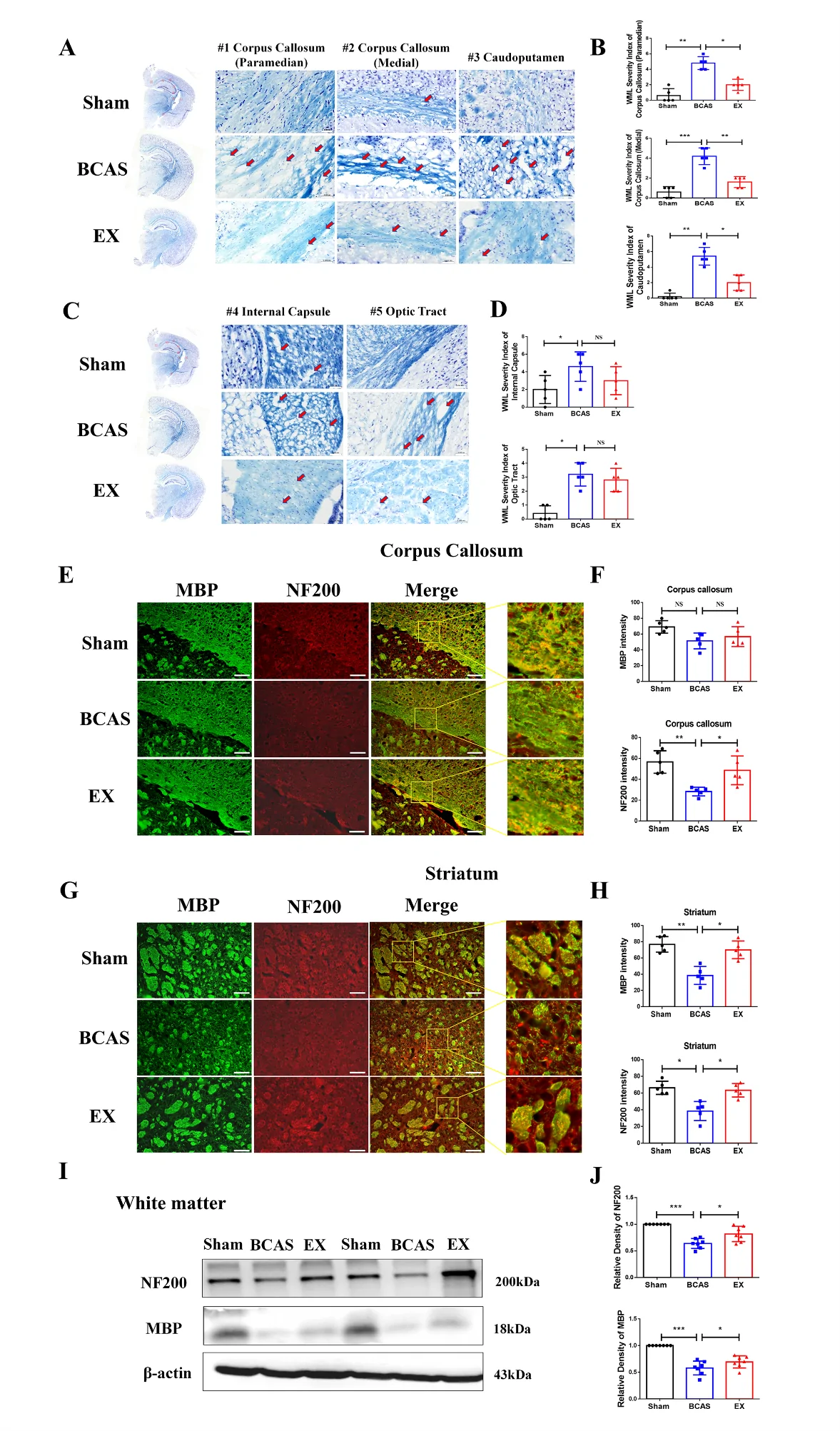
**Aerobic exercise increases WM integrity in BCAS mice**. (**A-D**) Representative Luxol Fast Blue (LFB)-stained images and corresponding quantification showing preserved white matter (WM) integrity across various regions: corpus callosum (paramedian and medial), caudoputamen, internal capsule, and optic tract. Scale bar: 50 µm. n = 5 mice per group. (**E**) Representative images of myelin basic protein (MBP, green) and neurofilament 200 (NF200, red) in the corpus callosum. (**F**) Immunofluorescence densitometric quantification of MBP and NF200 in the corpus callosum. (**G**) Representative images of MBP (green) and NF200 (red) in the striatum. (**H**) Immunofluorescence densitometric quantification of MBP and NF200 in the striatum. Scale bar: 1000 µm. n = 5 mice per group. (**I**) Representative western blot bands showing MBP and NF200 from WM samples. (**J**) Quantitative analysis of MBP and NF200 expression levels. Bar graphs are presented as mean ± SD. n = 7 mice per group. NS: no significance. *p < 0.05, **p < 0.01, ***p < 0.001.

Aerobic exercise significantly ameliorated these deficits in the EX group, reducing escape latency (p < 0.05, Fig. 1B, C) and increasing exploration time in the Y-maze (p < 0.001, Fig. 1F), suggesting improvements in hippocampal function and spatial memory. Despite no significant changes in the NOR test (p > 0.05, Fig. 1G), the findings indicate that aerobic exercise enhances cognitive performance by improving both hippocampal and possibly cortical functions. Balance beam tests across all groups showed no significant differences in post-surgical motility (p > 0.05, Fig. 1H), confirming that cognitive improvements were not confounded by changes in motor abilities. Laser speckle imaging revealed significant decrements in CBF post-BCAS modeling, with partial recovery by day 30 (p < 0.01, Fig. 1I, J), but no significant CBF restoration differences between the EX and BCAS groups two months post-modeling (p > 0.05, Fig. 1K, L). These results suggest that the spatial memory improvement in BCAS mice from aerobic exercise did not significantly affect CBF restoration.

### Aerobic Exercise Attenuates WM Damage in BCAS Mice

To explore how aerobic exercise enhances spatial memory in BCAS mice, the integrity of WM bundles was assessed using LFB staining and the expression of MBP and NF200. Compared to the Sham group, WM integrity in regions like the corpus callosum (paramedian and medial), caudoputamen, internal capsule, and optic tract was disrupted in the BCAS group. Aerobic exercise notably restored WM integrity in the corpus callosum (paramedian and medial) and caudoputamen after BCAS modeling (p < 0.05, Fig. 2A, B), though no significant improvements were seen in the internal capsule and optic tract (p > 0.05, Fig. 2C, D). Immunostaining and western blot analyses revealed significant increases in MBP and NF200 expression in the corpus callosum and striatum of the EX group (p < 0.05, Figs. 2E-I), demonstrating that aerobic exercise mitigates BCAS-induced WM demyelination and damage, thereby enhancing WM integrity and cognitive function.

### Aerobic Exercise Enhances Hippocampal Plasticity in BCAS Mice

**Figure 3.**
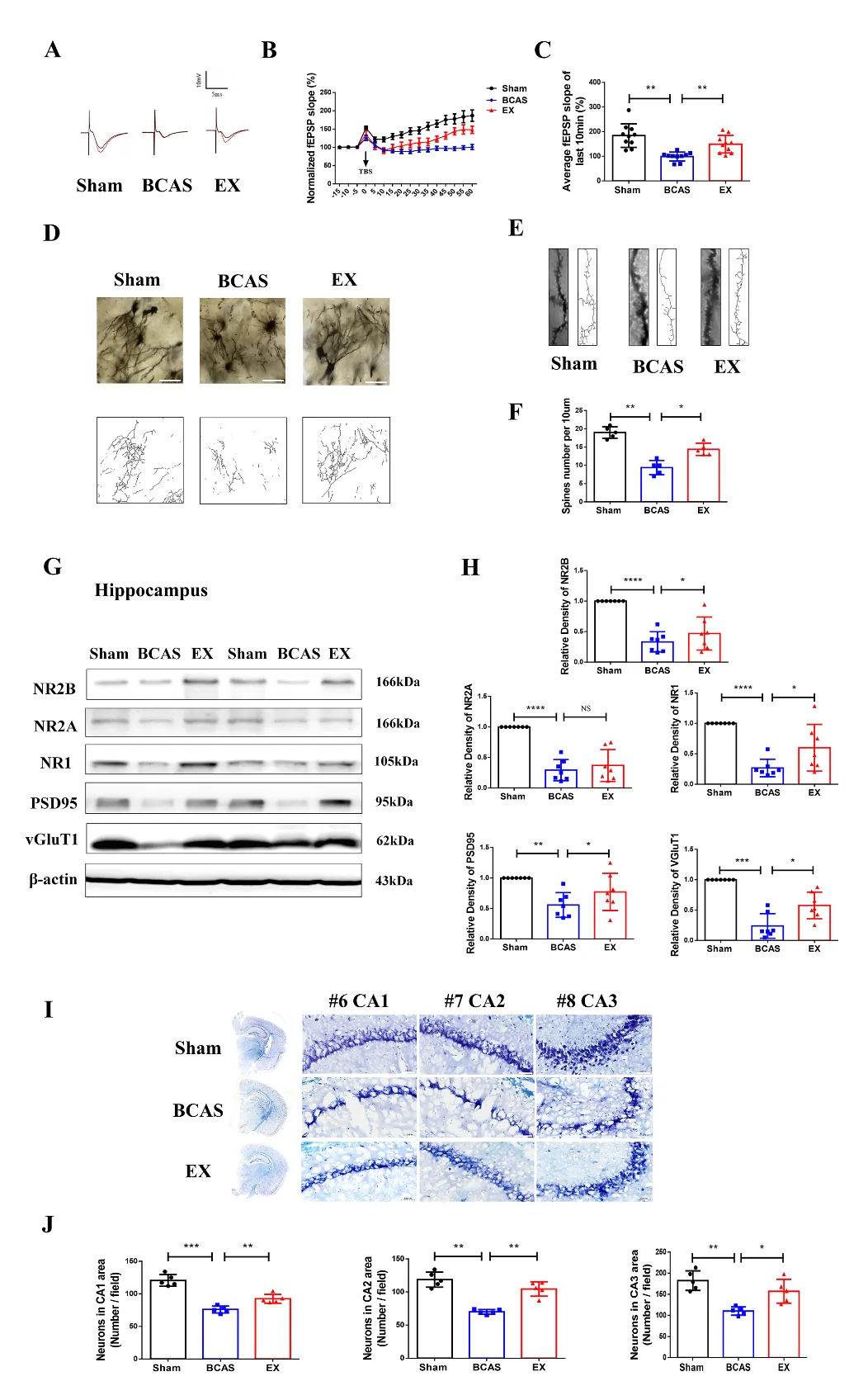
**Aerobic exercise enhances hippocampal synaptic plasticity in BCAS mice**. (**A**) Representative traces of field excitatory postsynaptic potentials (fEPSPs) before (black curve) and after (red curve) theta-burst stimulation (TBS) for long-term potentiation (LTP) induction. (**B**) Standardized fEPSP slope (%) with TBS indicated by black arrows. (**C**) Quantitative analysis of the mean slope of fEPSPs during the last 10 minutes of LTP recording. n = 10 slices from 5 mice. (**D**) Representative images of synaptic Golgi staining of neurons in the hippocampal region, including skeletonization images. (**E**) Representative image of dendritic spines. (**F**) Quantification of the number of dendritic spines per 10 µm. (**G**) Representative western blot bands for NR2B, NR2A, NR1, PSD95, and vGluT1 from hippocampal synaptosomes. (**H**) Quantitative analysis of NR2B, NR2A, NR1, PSD95, and vGluT1 expression levels. n = 7 mice per group. (**I**) Representative cresyl violet staining images of the hippocampal CA1, CA2, and CA3 regions. (**J**) Quantitative results of neurons in the hippocampal CA1, CA2, and CA3 regions. n = 5 mice per group. Bar graphs are presented as mean ± SD. NS: no significance. *p < 0.05, **p < 0.01, ***p < 0.001.

Synaptic plasticity in the hippocampus, crucial for spatial learning and memory, was assessed using the slope of excitatory postsynaptic potential (fEPSP) as an index of LTP. LTP was significantly reduced in the BCAS group compared to the Sham group, indicating impaired synaptic plasticity. Aerobic exercise significantly reversed this LTP decay in the EX group (p < 0.01, Fig. 3A-C). Golgi staining and dendritic spine counts showed significant increases in dendritic crossings and spine numbers in the EX group compared to the BCAS group (p < 0.05, Fig. 3D-F). The expression of synapse-associated proteins and N-Methyl-D-Aspartate (NMDA) receptor subunits was also evaluated. Notably, aerobic exercise mitigated the loss of these proteins in the EX group (p < 0.05, Fig. 3G, H). Cresyl violet staining quantified hippocampal neurons, revealing significant neuronal retention in the EX group compared to BCAS mice (p < 0.05, Fig. 3I, J). These results demonstrate that aerobic exercise enhances hippocampal plasticity, improving synaptic function, restoring synapse-associated proteins, and increasing neuronal survival.

### Aerobic Exercise Increases Astrocyte Production and A2 Phenotype Transformation

**Figure 4.**
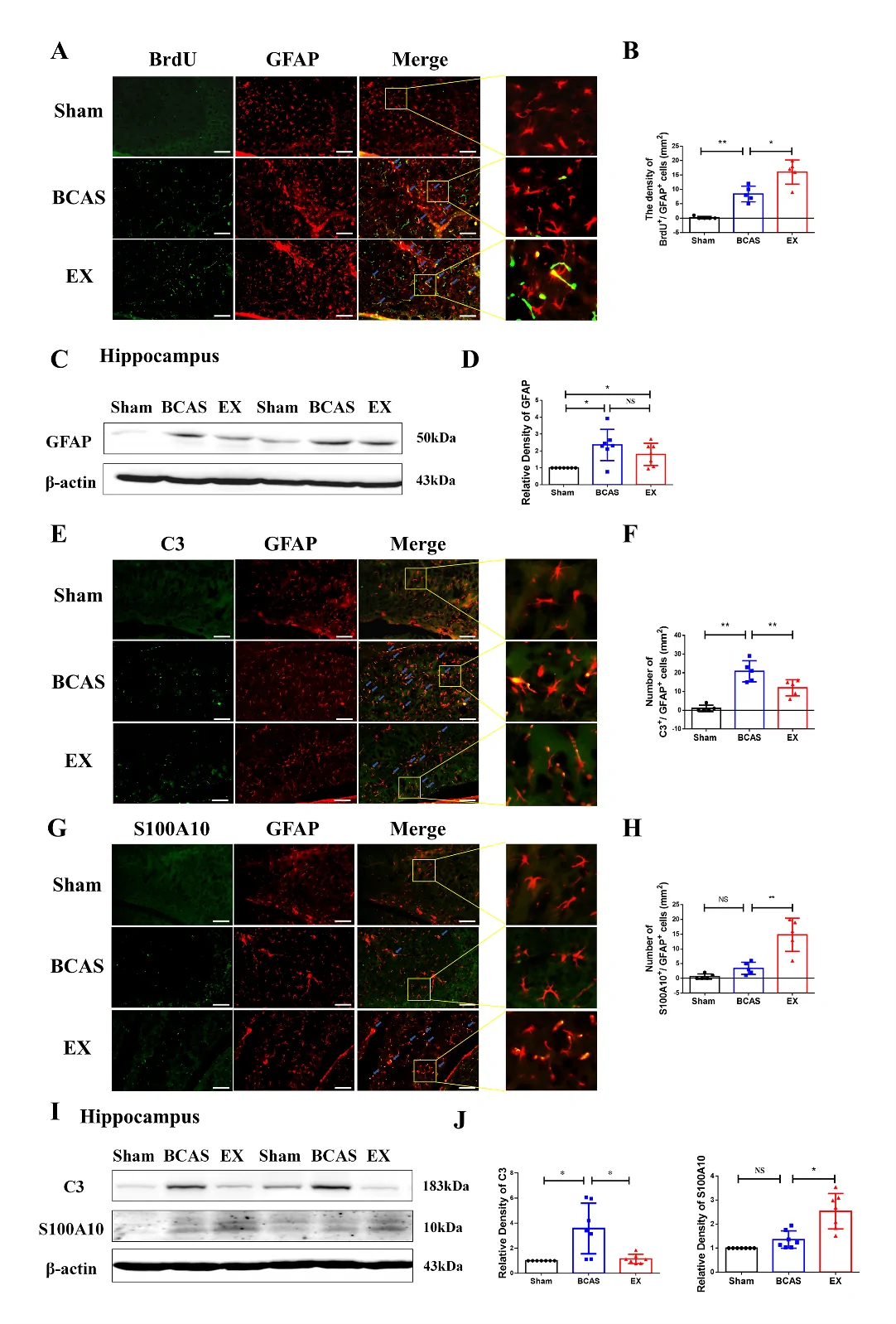
**Aerobic exercise increases astrocytogenesis and A2 phenotypic transformation in BCAS mice**. (**A**) Representative images showing BrdU (green, neoplastic cells) and GFAP (red, astrocytes). Scale bar, 100 µm. (**B**) Quantification of BrdU^+^/GFAP^+^ cells per square millimeter. n = 5 mice per group. (**C**) Representative western blot bands of GFAP from hippocampal tissue. n = 7 mice per group. (**D**) Quantitative analysis of GFAP expression levels. (**E**) Representative images showing C3 (green, A1 phenotype astrocytes) and GFAP (red, astrocytes). Scale bar, 100 µm. (**F**) Quantification of C3^+^/GFAP^+^ cells per square millimeter. n = 5 mice per group. (**G**) Representative images showing S100A10 (green, A2 phenotype astrocytes) and GFAP (red, astrocytes). Scale bar, 100 µm. (**H**) Quantification of S100A10^+^/GFAP^+^ cells per square millimeter. n = 5 mice per group. (**I**) Representative western blot bands of C3 and S100A10 from hippocampal tissue. (**J**) Quantitative analysis of C3 and S100A10 expression levels. n = 7 mice per group. Bar graphs are presented as mean ± SD. NS: no significance. *p < 0.05, **p < 0.01, ***p < 0.001.

Astrocyte proliferation, a significant pathology in BCAS-induced injury, was evident with increased numbers of proliferating astrocytes (BrdU and GFAP co-stained) in both BCAS and EX groups compared to Sham (p < 0.05, Fig. 4A-D). Aerobic exercise significantly promoted the transformation from pro-inflammatory A1 to anti-inflammatory A2 astrocytes, as demonstrated by decreased expression of complement component C3 and increased S100A10 in the EX group (p < 0.01, Figs. 4E-H), further supported by western blot analysis (p < 0.05, Fig. 4I, J). This suggests that aerobic exercise enhances anti-inflammatory responses through astrocytic phenotype modulation, contributing to the protective effects against cerebral injury.

### Aerobic Exercise Increases ANXA1 Expression and Inhibits the MAPK Signaling Pathway Activated by BCAS

**Figure 5.**
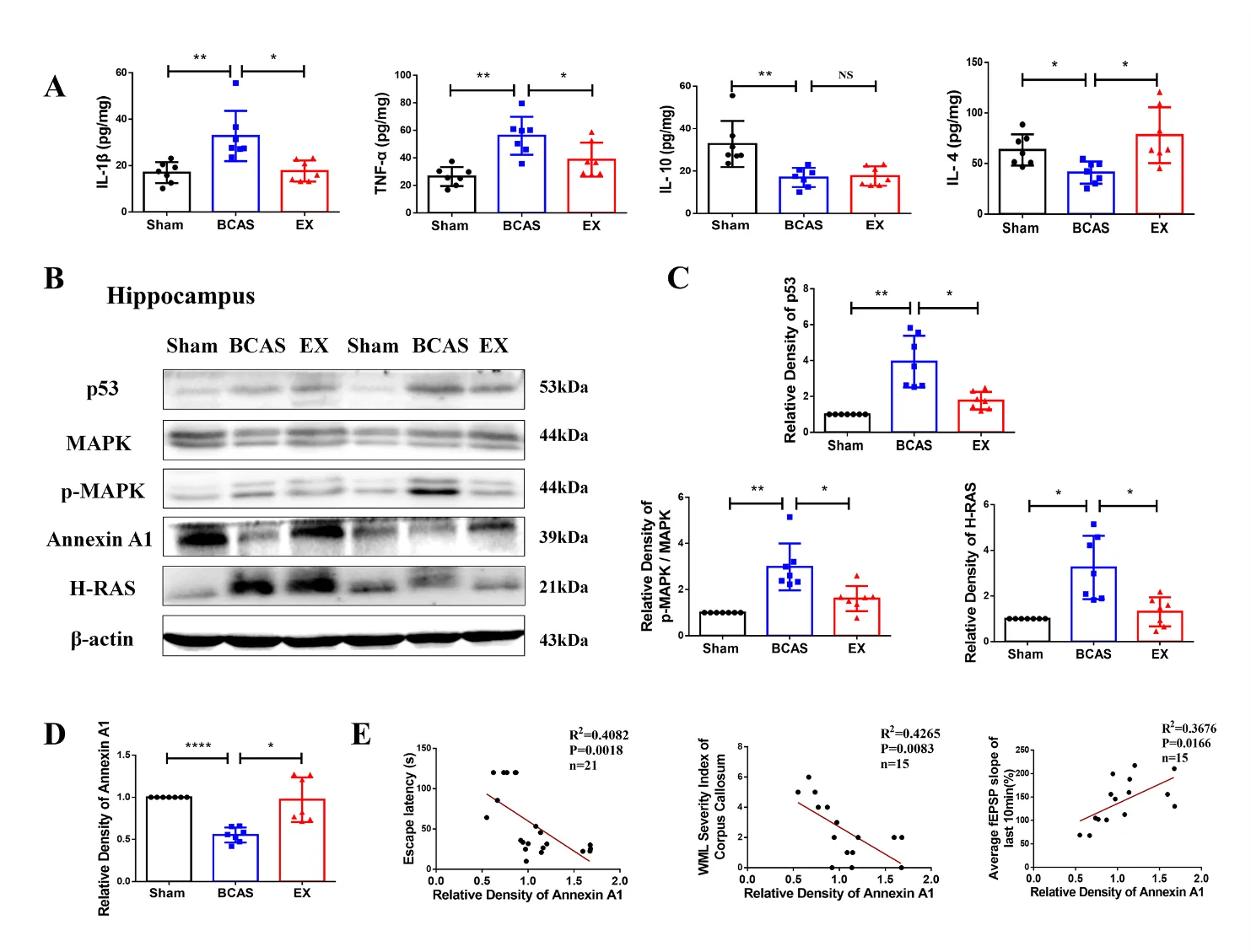
**Aerobic Exercise Increases ANXA1 Expression and Inhibits MAPK Signaling in BCAS Mice**. (**A**) Quantification of inflammatory cytokines (IL-1β, IL-4, IL-10, TNF-α) using ELISA. (**B**) Representative western blot bands for p53, MAPK, phosphorylated MAPK (p-MAPK), ANXA1, and H-RAS from hippocampal tissue. (**C**) Quantitative analysis of the expression levels of p53, p-MAPK/MAPK, and H-RAS. (**D**) Quantitative analysis of ANXA1 expression levels. n = 7 mice per group. (**E**) Pearson correlation analysis between the relative density of ANXA1 and escape latency, ANXA1 and white matter (WM) severity index, as well as between ANXA1 and field excitatory postsynaptic potentials (fEPSPs). Bar graphs are presented as mean ± SD. NS: no significance. *p < 0.05, **p < 0.01, ***p < 0.001.

Systemic inflammatory status was analyzed by measuring serum levels of inflammatory cytokines. Pro-inflammatory cytokines IL-1β and TNF-α were significantly elevated in the BCAS group compared to Sham, while aerobic exercise markedly reduced their expression and increased the anti-inflammatory cytokine IL-4 in the EX group (p < 0.05, Fig. 5A). ANXA1 expression, decreased in the BCAS group, was restored by aerobic exercise (p < 0.05, Figs. 5B, D). The MAPK signaling pathway, critical for neuroinflammation and reactive astrocyte activation, showed suppressed protein expression following aerobic exercise (p < 0.05, Figs. 5B, C). Correlation analyses revealed significant associations between ANXA1 expression and improvements in cognitive function, WM integrity, and hippocampal synaptic plasticity (p < 0.001, Fig. 5E). These findings suggest that ANXA1 plays a pivotal role in mediating the beneficial effects of aerobic exercise on spatial memory and WM integrity.

### Exercise-Derived Exosomes Enhance ANXA1 Expression and Inhibit MAPK Signaling Pathway

**Figure 6.**
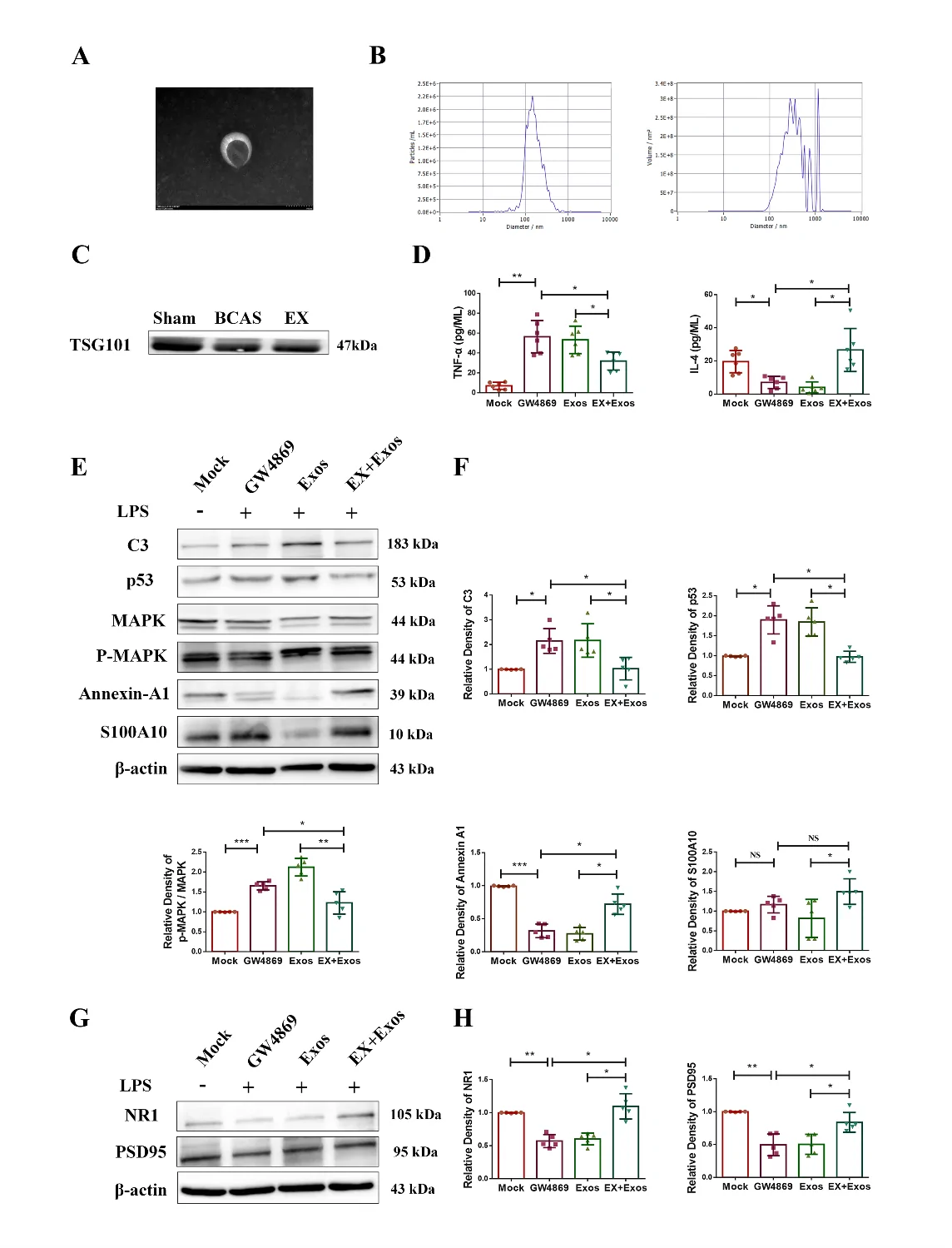
**Exercise-Derived Exosomes Enhance ANXA1 Expression and Inhibit MAPK Signaling in a Co-Culture Model**. (**A**) Representative transmission electron microscopy (TEM) images displaying the morphology of exosomes. (**B**) Diameter and volume of exosomes measured using nanoparticle tracking analysis (NTA). (**C**) Western blot detection of exosomes using the positive marker TSG101. (**D**) Quantification of inflammatory cytokines (TNF-α and IL-4) using ELISA. (**E**) Representative western blot bands showing C3, p53, MAPK, phosphorylated MAPK (p-MAPK), ANXA1, and S100A10 from U251 astrocyte cells. (**F**) Quantitative analysis of the expression levels of C3, p53, MAPK, p-MAPK, ANXA1, and S100A10. (**G**) Representative western blot bands for PSD95 and NR1 from HT22 neuronal cells. (**H**) Quantitative analysis of PSD95 and NR1 expression levels. n = 5 mice per group. Bar graphs are presented as mean ± SD. NS: no significance. *p < 0.05, **p < 0.01, ***p < 0.001.

A co-culture model of U251 astrocytes and HT22 neurons was established to further investigate the relationship between exercise and the ANXA1/MAPK axis. Lipopolysaccharide (LPS) induced cell injury was modulated by exosomes extracted from mouse brain tissue. Exercise-derived exosomes significantly reduced pro-inflammatory TNF-α expression and increased anti-inflammatory IL-4 in the co-culture (p < 0.05, Fig. 6D). Quantitative protein analysis showed that exercise exosomes attenuated LPS-induced increases in the pro-inflammatory marker C3 while enhancing the expression of the anti-inflammatory marker S100A10 (p < 0.05, Fig. 6E, F). In HT22 neurons, exercise-secreted exosomes increased the expression of synapse-associated proteins PSD95 and NR1, suggesting improved synaptic function and recovery following LPS-induced injury (p < 0.05, Fig. 6G, H). These results indicate that aerobic exercise promotes anti-inflammatory responses and synaptic repair by modulating the ANXA1/MAPK axis through exosome-mediated communication, contributing to enhanced synaptic plasticity and neuronal recovery in the context of inflammatory injury.

### DISCUSSION

**Figure 7.**
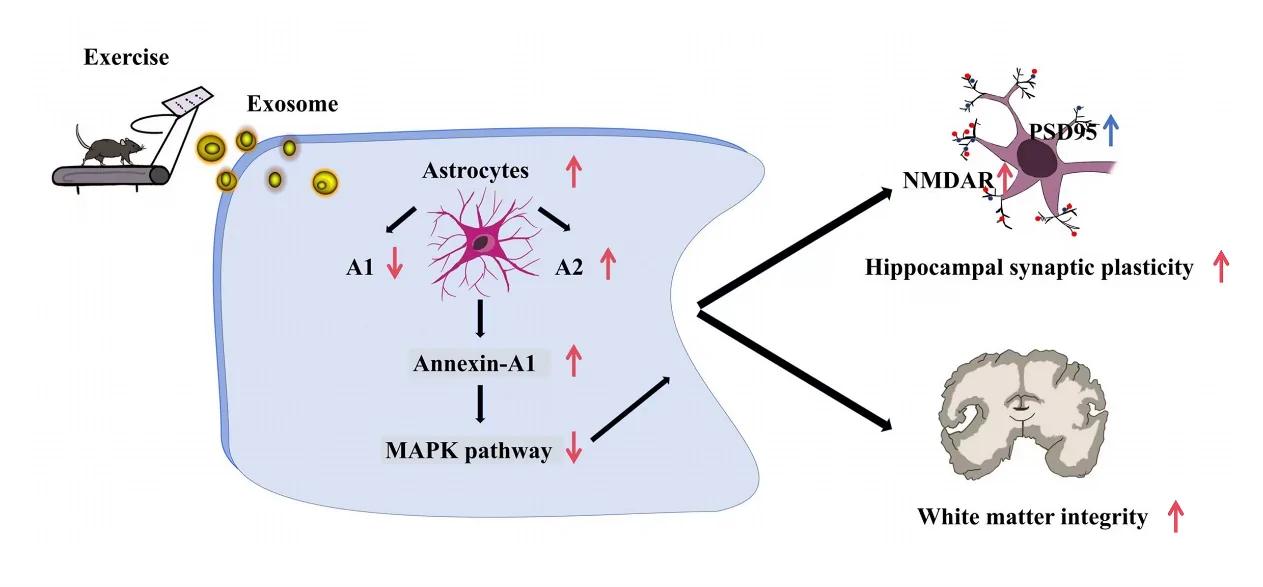
**Diagram Illustrating the Impact of Aerobic Exercise on the ANXA1-MAPK Axis Through Secreted Exosomes in BCAS Mice**. This diagram details how exosomes derived from aerobic exercise crucially modulate the central immune-inflammatory response, primarily via astrocyte-mediated mechanisms. Notably, type A2 astrocytes, which are promoted by these exosomes, enhance ANXA1 expression and concurrently inhibit the MAPK signaling pathway, typically activated during BCAS. These interactions collectively bolster hippocampal plasticity and white matter integrity, thus contributing to the alleviation of cognitive impairments associated with chronic cerebral hypoperfusion.

The main findings of this study reveal several impactful outcomes of aerobic exercise on mice with chronic cerebral hypoperfusion. Firstly, aerobic exercise significantly enhances spatial memory by improving WM integrity and hippocampal plasticity. Secondly, it increases the expression of the endogenous mediator ANXA1, which subsequently inhibits the BCAS-induced activation of the MAPK signaling pathway. Lastly, the exosomes secreted during aerobic exercise are found to improve astrocyte-mediated central immune responses and synaptic plasticity through the ANXA1/MAPK axis, as illustrated in Figure 7. These results underscore the multifaceted benefits of aerobic exercise in the neurological rehabilitation of mice, suggesting potential therapeutic insights for similar conditions in humans.

In recent years, the beneficial effects of aerobic exercise have been increasingly recognized across various types of dementia. For Alzheimer's disease (AD), it has been shown to enhance cognitive function by modulating microglial glucose metabolism and promoting hippocampal plasticity [43]. Similarly, aerobic exercise has improved cognitive performance in mixed dementia and Parkinson’s disease dementia by boosting the expression of neurotrophic factors [44]. Our findings contribute to this body of knowledge by demonstrating that aerobic exercise enhances spatial memory capacity in BCAS mice through improvements in WM integrity and hippocampal function. This aligns with previous research, supporting the idea of aerobic exercise as a multifaceted intervention for neurological health.

ANXA1, known for its anti-inflammatory properties, has been studied in conditions such as ulcerative colitis, diabetic nephritis, and allergic airway inflammation [45-47]. However, its role in VCI has been less explored. This study provides new evidence that aerobic exercise upregulates ANXA1 expression in brain tissue, reversing the significant reductions caused by BCAS. Furthermore, aerobic exercise has been observed to decrease pro-inflammatory cytokines (IL-1β, TNF-α) and increase anti-inflammatory cytokines (IL-4, IL-10) in the peripheral blood serum of mice, suggesting that aerobic exercise enhances the systemic anti-inflammatory response mediated by ANXA1 in the BCAS model.

Astrocytes, crucial to the innate immunity of the central nervous system, are increasingly recognized for their role in improving motor-related cognitive functions [48]. Understanding how aerobic exercise influences astrocyte responses, especially the transitions between neurotoxic A1 and neuroprotective A2 phenotypes, is vital for elucidating the neuroinflammatory and neuroprotective mechanisms of exercise. It is well established that neural stem cell neogenesis in adult brain tissue primarily occurs in the subventricular zone (SVZ) of the lateral ventricles and the subgranular zone (SGZ) of the dentate gyrus in the hippocampus [49]. These stem cells can differentiate into neurons, astrocytes, and oligodendrocytes, each fulfilling distinct roles [50]. Our study observed an increase in astrocyte neogenesis, particularly within the hippocampal SGZ region, following aerobic exercise. Notably, there was a significant increase in the anti-inflammatory A2 astrocyte phenotype in the exercise group compared to the BCAS group, indicating that the newly generated astrocytes exhibited enhanced anti-inflammatory properties.

The MAPK pathway plays a crucial role as a regulatory gene for reactive astrocytes, with significant involvement in brain injury and as a major regulator of neuroinflammation [52]. In our study, BCAS was observed to activate MAPK signaling-related proteins such as p53, p-MAPK/MAPK, and H-RAS, while aerobic exercise effectively inhibited this signaling activity. Recent research supports the notion that MAPK may operate downstream of ANXA1 [53]. Our findings further suggest that activation of ANXA1 by exercise not only promotes astrocyte proliferation but also encourages a shift towards an anti-inflammatory phenotype by inhibiting the MAPK signaling pathway.

Evidence strongly suggests that astrocyte polarization significantly affects WM function [54]. In conditions such as cerebral ischemia, astrocytic AQP4-mediated brain edema is a primary cause of high WM signal intensity [55, 56]. Considering that WM primarily consists of myelin and axons essential for neural signaling [57], the chronic cerebral hypoperfusion typical of BCAS can lead to demyelination and axonal damage within WM [58]. Our study demonstrated that aerobic exercise aids in repairing this WM damage, highlighting the integral role of astrocytes in maintaining neuronal function throughout the nervous system. Astrocytes supply lactate as an energy source and are instrumental in regulating synaptic maturation and neurotransmitter release in hippocampal neurons [60]. They are crucial for synapse formation, maturation, and maintenance [61], which this study hypothesized to enhance through aerobic exercise.

Neurophysiological assessments revealed that BCAS diminished excitatory postsynaptic potentials, whereas aerobic exercise significantly increased them. Moreover, aerobic exercise counteracted the BCAS-induced reduction in glutamate delivery protein vGluT1 and synaptic cadherin proteins PSD95. Given the importance of the CA1, CA2, and CA3 regions of the hippocampus for memory encoding and retrieval [62], preserving hippocampal neurons is vital for cognitive function. Our results confirm that aerobic exercise mitigates hippocampal neuronal loss induced by BCAS, thereby protecting hippocampal synaptic plasticity. Notably, NMDA receptors, which are critical therapeutic targets for cognitive disorders [64], showed enhanced expression of NR2B and NR1 subunits, and increased interaction with the scaffolding protein PSD95 due to exercise-induced mechanisms.

Furthermore, our research suggests that exercise-induced exosomes play a pivotal role in intercellular communication and contribute to disease modulation. These exosomes, enriched in molecular signals, have been shown to regulate immune-inflammatory responses in vivo via the ANXA1/MAPK axis and enhance synaptic plasticity in cultured neuronal cells [65]. This aligns with findings that exosomes from trained athletes can effectively manage age-related metabolic disorders [66], underscoring the therapeutic potential of exercise-derived biological vectors.

Notably, while aerobic exercise improved cognitive function and WM integrity in BCAS mice, it did not significantly enhance CBF recovery. This observation suggests that the cognitive improvements may stem from alternative mechanisms, such as the neuroprotective effects of exercise on synaptic plasticity and astrocyte function, rather than from the restoration of blood flow alone. Additionally, no significant differences were observed in the NOR discrimination index between the EX and BCAS groups, indicating that aerobic exercise selectively enhanced spatial memory without significantly improving nonspatial recognition memory.

Our study lays a foundation for non-pharmacological approaches to treating VCI. For clinical translation, further in vitro studies are necessary to evaluate the effects of aerobic exercise-derived exosomes on ANXA1 expression and MAPK signaling. Expanding experimental subgroups to examine the impact of varying exercise intensities on biomarkers identified in animal models, such as ANXA1 and inflammatory cytokines, could provide a basis for early-phase clinical trials.

There are also some limitations to this study. A moderate-intensity exercise model was adopted based on preliminary research; however, further investigations are needed to explore a wider range of exercise intensities, durations, and modes. Additionally, while this study underscores the significant role of exercise-derived exosomes in disease models, the specific bioactive components within these exosomes remain unidentified and warrant further elucidation.

### Conclusions

Astrocytes play a pivotal role in neuronal support and synaptic function. Our study underscores the potential of aerobic exercise to influence astrocyte phenotype transformation, promoting neuroprotection and enhancing cognitive functions. By mapping the interaction between exercise-induced exosomes and neuroinflammatory pathways, we offer a foundational perspective for non-pharmacological approaches to treat VCI. Future studies should further explore the impact of different exercise intensities and durations on ANXA1 expression and inflammatory markers, paving the way for early-phase clinical trials.

This research also highlights the need for a broader understanding of the bioactive components within exercise-derived exosomes, which could inform targeted therapies for neurodegenerative diseases. While the study is robust in its findings, further investigations into the range of exercise modalities and their direct and indirect effects on cognitive health are essential.
